# Evaluation of the truebeam machine performance check (MPC) geometric checks for daily IGRT geometric accuracy quality assurance

**DOI:** 10.1002/acm2.12064

**Published:** 2017-03-22

**Authors:** Michael P Barnes, Peter B Greer

**Affiliations:** ^1^ Department of Radiation Oncology Calvary Mater Hospital Newcastle NSW 2298 Australia; ^2^ School of Medical Radiation Sciences University of Newcastle Newcastle NSW 2308 Australia; ^3^ School of Mathematical and Physical Sciences University of Newcastle Newcastle NSW 2308 Australia

**Keywords:** linac quality assurance, machine performance check (MPC)

## Abstract

Machine Performance Check (MPC) is an automated and integrated image‐based tool for verification of beam and geometric performance of the TrueBeam linac. The aims of the study were to evaluate the performance of the MPC geometric tests relevant to OBI/CBCT IGRT geometric accuracy. This included evaluation of the MPC isocenter and couch tests. Evaluation was performed by comparing MPC to QA tests performed routinely in the department over a 4‐month period. The MPC isocenter tests were compared against an in‐house developed Winston–Lutz test and the couch compared against routine mechanical QA type procedures. In all cases the results from the routine QA procedure was presented in a form directly comparable to MPC to allow a like‐to‐like comparison. The sensitivity of MPC was also tested by deliberately miscalibrating the appropriate linac parameter. The MPC isocenter size and MPC kV imager offset were found to agree with Winston–Lutz to within 0.2 mm and 0.22 mm, respectively. The MPC couch tests agreed with routine QA to within 0.12 mm and 0.15°. The MPC isocenter size and kV imager offset parameters were found to be affected by a change in beam focal spot position with the kV imager offset more sensitive. The MPC couch tests were all unaffected by an offset in the couch calibration but the three axes that utilized two point calibrations were sensitive to a miscalibration of the size in the span of the calibration. All MPC tests were unaffected by a deliberate misalignment of the MPC phantom and roll of the order of one degree.

## 1 Introduction

In external beam radiotherapy the aim is to accurately deliver a prescribed radiation dose to a predefined target volume, while minimizing dose outside the target. The associated geometric aspect of this process is continually being developed and the accuracy improved. It is now common practice to align and shift patients based upon images of the patient taken from imaging systems integrated into the linac. The most common form of this image‐guided radiotherapy (IGRT) is with kiloVoltage (kV) imaging systems aligned to the linac isocenter. Such systems can be used as either planar x rays [On‐Board‐Imager (OBI)] or as Cone‐Beam Computed Tomography (CBCT).^1^ The correct functioning and accuracy of these systems are paramount for the geometrically accurate delivery of the treatment 2, 3, 4, 5, 6 and the required accuracy is increasing as treatment margins are reduced to spare healthy tissue.

Daily Quality Assurance (QA) testing of Linear Accelerator (linac) IGRT functionality is recommended in the AAPM Task Group report 179,^7^ which was published in 2012 with specific QA recommendations for CT‐based IGRT technologies. TG‐179 recommends daily QA testing of collision and other interlocks, warning lights, laser/image/treatment isocenter coincidence or repositioning with couch shift. The isocenter coincidence and repositioning tests have a recommended tolerance of ± 2 mm.

With the TrueBeam 2.0 platform Varian (Varian Medical Systems, Palo Alto, CA, USA) has released the Machine Performance Check (MPC) application. MPC is a fully integrated image‐based tool for assessing the performance of the TrueBeam critical functions on a daily basis. MPC tests are based upon EPID and OBI images with and without the IsoCal phantom and the tests are split into two categories: The beam constancy checks and the geometric checks. It is the geometric tests specific to the OBI/CBCT systems which are the focus of this study. EPID‐based linac daily QA has been performed previously by Sun et al., 2015.^8^ However, the tests evaluated by Sun et al. are different to those presented in this study.

At the time of writing there were only two papers in the literature pertaining to evaluation of MPC. Clivio et al., 2015 9 published work whereby the results of MPC were compared against other more standard QA techniques. In this study, both MPC and the standard QA tests were run together on 10 consecutive days. From this dataset the mean and standard deviation was calculated for both MPC and standard QA measurements and compared. The short duration of the study does not allow for any assessment of long‐term stability and there is no measure of MPC sensitivity, both of which are acknowledged by the authors. More recently, Barnes and Greer 10 published work, whereby the MPC beam constancy checks were further evaluated building on the work of Clivio et al.

It is the aim of this study to compare the MPC geometric checks that are relevant to the OBI/CBCT system against standard QA tests to provide an evaluation of MPC as an IGRT geometric QA device. The study was performed over a longer period (4 months) than the Clivio study and provides an assessment of the MPC stability and sensitivity to drift of the linac systems being tested. Sensitivity is further examined by the use of deliberate changes to both offset and span of the couch calibrations and for the isocenter tests to an offset in the beam focal spot position. The study attempts to provide standard QA results in a form that is directly comparable to the equivalent MPC test. This study evaluates different MPC checks than those evaluated by Barnes and Greer 10 and hence the two bodies of work complement each other toward evaluating the whole of MPC as a linac QA device.

## 2 Methods

All measurements in this study were performed on a single Varian TrueBeam STx (software version 2.0) linac fitted with an aS1200 EPID and six degree of freedom couch.

### 2.A Materials

#### 2.A.1 MPC Geometric checks

The MPC geometric tests utilize a series of kV and 6 MV beam images of the IsoCal phantom situated in a specific bracket on the IGRT couch top to assess: treatment/radiation isocenter size, MV and kV imager center pixel offsets from projected radiation isocenter, accuracy of collimator and gantry angles, accuracy of jaw and MLC leaf positions and accuracy of couch positioning including pitch and roll. All measurements are highly automated and the user is simply required to setup the IsoCal phantom and bracket onto the treatment couch at position H2 and to beam‐on for each required energy. For the geometric tests the system makes all required motions automatically and beams on when all is in position. Images are automatically analyzed at the TrueBeam console and results are presented with a nonuser definable pass/fail criteria applied. Functionality for presenting trends in results is also available in the package. The relevant MPC checks to the Varian OBI/CBCT IGRT systems are the isocenter size, kV imager offset, and the couch tests.

#### 2.A.2 Winston–Lutz

The radiation isocenter position and size has been traditionally tested using the Winston–Lutz test.^11^ For routine QA the department uses a variant of the Winston–Lutz test developed by Rowshanfarzad et al., 2011,^12^ whereby the field is defined by a stereotactic cone and MV images are taken of a ball bearing that has been prepositioned at the imaging isocenter. Imaging is performed using the EPID in cine acquisition mode while a conformal gantry arc, collimator. or couch rotation is performed. An in‐house developed MATLAB script (The Mathworks Inc., Natick, MA, USA) is used to calculate the position of the ball bearing compared to the center of the field for each EPID cine frame to allow calculation of the mean and maximum displacement of the ball bearing and hence imaging isocenter from the radiation field center defined by the stereotactic cone.

### 2.B Measurement methods

#### 2.B.1 Repeatability

Short‐term repeatability of the MPC geometric tests was evaluated by taking five successive measurements and calculating the standard deviation.

#### 2.B.2 Isocenter

The radiation isocenter centroid is the ideal intersection point of the beams central axis over a full gantry rotation. The central beam axis in MPC is defined by the center of rotation of the MLC. This is measured using EPID images of open MLC defined fields at eight representative gantry angles 45° apart. At each gantry angle two images are taken with 180 degree‐opposed collimator angles to determine the beam central axis independent of MLC positional accuracy. In MPC, the size of the radiation isocenter spheroid is defined as the maximum distance of the beam's central axis from the idealized isocenter centroid.^13^ The MPC isocenter size parameter reported is a single value, which means that no information is available on isocenter shape or in which direction the isocenter has deviated the most from the centroid. The MPC isocenter size tolerance is set at ± 0.5 mm. Besides isocenter size, MPC also reports the kV and MV imager offset. These parameters represent the maximum distance of the imager center from the projection of the radiation isocenter centroid. These parameters are included to provide a measure of the correctness of the IsoCal calibration, which is important for aligning the radiation and imaging isocenters and for CBCT image quality.

##### *Isocenter size*

The in‐house Winston–Lutz analysis program reports both the maximum and mean measured deviation of the radiation field center of the cone from the ball bearing. The initial setup of the ball bearing using cone‐beam CT places it at the estimated centroid of imaging isocenter. Results are presented in the plane of the EPID (scaled to isocenter distance) for both the panel inplane and “crossplane” directions. In this method, the mean deviation parameter represents the distance between the centroids of imaging and radiation isocenters and the maximum deviation represents the greatest distance between any point within the radiation isocenter and the centroid of imaging isocenter. By calculating the difference between measured maximum deviation and mean deviation and then calculating the vector magnitude from the inplane and crossplane components, the maximum size of the radiation isocenter spheroid is determined and is then directly compared to the MPC isocenter size parameter.

##### *kV imager offset*

For accurate IGRT the imaging system and radiation isocenters must coincide.^7^ This is achieved on Varian linacs using the IsoCal calibration procedure,^14^ which has been validated by Gao et al., 2014 15 and by Chiu et al., 2015.^16^ The kV and MV systems share a common axis of gantry rotation. On the TrueBeam linac, the IsoCal procedure aligns the radiation and imaging isocenters by adjustment of kV detector panel position in both lateral and longitudinal directions during beam‐on such that at each gantry angle the center of the panel coincides with the projection of the radiation isocenter centroid. The IsoCal verification procedure reruns the IsoCal calibration procedure with IsoCal corrections applied and reports the maximum measured displacement between the radiation isocenter and the center of the kV imager panel. This provides a measure of the validity of the current IsoCal calibration. In MPC, the kV imager offset parameter provides a similar measure of the validity of the current IsoCal calibration and hence is a surrogate for the coincidence of radiation and imaging isocenters.

The kV imager offset reported by MPC was compared against the in‐house Winston–Lutz method. The in‐house Winston–Lutz mean deviation parameter is the distance between imaging and radiation isocenter centroids. The software reports this parameter for both the EPID crossplane and inplane components. From these two components the vector magnitude was calculated and compared against MPC kV imager offset. In this comparison the Winston–Lutz method directly represents the radiation and imaging isocenter coincidence, while the MPC kV imager offset is a surrogate for radiation and imaging isocenter coincidence via the IsoCal calibration.

##### *Sensitivity of isocenter checks to focal spot position change*

The size and shape of the radiation isocenter is dependent on the size of the mechanical isocenter and the position of the beam focal spot relative to collimator rotation axis. During the data collection period for this study an adjustment was required to be made to the linac position steering servo balance point and hence the position of the focal spot. The position steering was adjusted such that a change in position of the beam profile of 0.4 mm was observed at isocenter plane. The MPC isocenter tests performed leading up to and post the adjustment were analyzed for sensitivity to this change.

#### 2.B.3 Couch

In the authors department couch readouts are tested by comparing the readout against an external measure at a few representative points. MPC does not report on the absolute couch positioning but rather on the measured distance traveled between two points. This is the most clinically important aspect of couch motion for ensuring that couch shifts based upon IGRT imaging are accurate. To allow a meaningful comparison between the MPC couch travel and the departmental couch tests, the departmental couch test results are presented in terms of difference between the two most extreme measurement points. This measured range is then scaled to the range over which the MPC travel range is measured. The difference of this measured range from expected is then compared to MPC.

##### *Couch Position tests*

In the authors department couch position checks are based upon the Varian Customer Acceptance Procedures.^17^ Couch vertical readouts are measured using calibrated front pointers over a 10 cm range (MPC range = 15 cm). Couch lateral is measured with calibration Lokbar™ and steel ruler over a 20 cm range (MPC range = 5 cm). Couch longitudinal is measured using calibration Lokbar and tape measure over a 90 cm range (MPC range = 5 cm). Couch angle is verified at cardinal angles using the cross hairs projected onto graph paper after aligning the cross hair with the axis of gantry rotation using the swing test method. This gives a measurement range of 180° (MPC range = 10°). The pitch and roll readouts for the six degree of freedom couch are checked against a digital spirit level rated to ± 0.01 degree accuracy (Digi‐Pas 2‐Axis Precision Digital level, DWL3000XY, DIGIPAS USA LLC) at 0 and ± 2° giving a 4° range (MPC range = 3°).

##### *Sensitivity to miscalibration*

In an experiment to test the sensitivity of the MPC couch results the couch was deliberately miscalibrated in each axis using the standard calibration procedures. This was done in two ways. Firstly, a systematic offset was introduced into the calibration. This was done to all couch axes individually using offsets of the magnitude similar to the MPC tolerance. Secondly, the span of the calibration was made successively both smaller and larger. The magnitude of the miscalibration was calculated to cause error at about MPC tolerance. The altered span miscalibration was performed only for couch lateral, longitudinal, and vertical. This was not possible for couch pitch, roll, and rotation because these utilized single point calibration procedures. In all cases the measured change in MPC was compared against expected from the miscalibration.

#### 2.B.4 Sensitivity of MPC to Phantom tilt

In an experiment to test the sensitivity of the MPC couch pitch and roll test to discrepancies in the phantom setup, the roll and pitch of the phantom were successively deliberately adjusted by approximately 1°. With the couch pitch and roll set to zero, the MPC bracket and phantom were attached to the couch top as per usual. The digital spirit level was used on the top surface of the phantom to measure the phantom pitch. The spirit level was then placed on the phantom handle as an initial measure of roll. MPC was then performed. The roll of the phantom was then adjusted by wedging paper sheets between the phantom and bracket until a change in roll was measured on the spirit level of one degree. MPC was repeated with the paper wedges in situ. The wedges were then removed and placed under the phantom to induce a change in pitch of one degree on the spirit level and MPC was again repeated. Any changes in MPC parameters between the three acquisitions were recorded.

## 3 Results

### 3.A Repeatability

The results of Table 1 show how repeatable each of the MPC geometric tests were across five successive measurements. The repeatability results of Table 1 show that for all MPC tests the methods are repeatable to within 0.05 mm or 0.04 degrees for all parameters at one standard deviation.

**Table 1:** Short term repeatability of the MPC isocenter and couch geometric tests based upon five successive measurements

Test	Standard Deviation
Couch	
Lateral	0.04 (mm)
Longitudinal	0.02 (mm)
Vertical	0.02 (mm)
Pitch	0.01 (Degrees)
Roll	0.00 (Degrees)
Rotation	0.01 (Degrees)
Isocenter	
kV offset	0.05 (mm)
size	0.02 (mm)

### 3.B Isocenter

#### 3.B.1 Isocenter size

**Figure 1:**
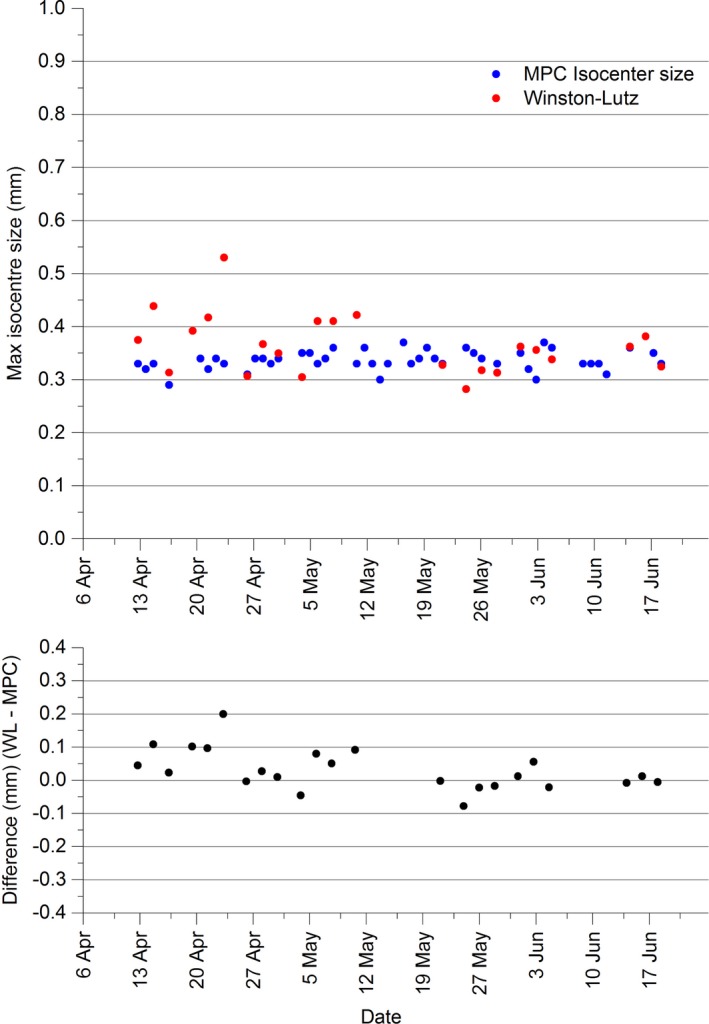
Comparison between isocenter sizes as measured using MPC and in‐house Winston–Lutz.

The results of Fig. 1 show that the MPC measured isocenter size ranged between 0.29 mm and 0.37 mm over the period. No drift in the results was detected so a calculation of the mean and standard deviation was performed and found to be 0.34 ± 0.02 mm (1 SD). Over the period there was greater variation in the Winston–Lutz results with data falling in the range 0.28 to 0.53 mm with mean of 0.37 ± 0.06 mm (1 SD). Results of the t‐test indicate that the two methods are not in statistical agreement [t(24)=2.42, *P* = <0.024], however, the MPC mean is within the 95% confidence interval [0.34, 0.39] of the Winston–Lutz mean. For judging the clinical significance of the differences between the two methods, Fig. 1 shows that MPC and Winston–Lutz were always within agreement within ± 0.2 mm and if the single outlying data point is removed then agreement is always within ± 0.11 mm.

#### 3.B.2 kV imager offset

**Figure 2:**
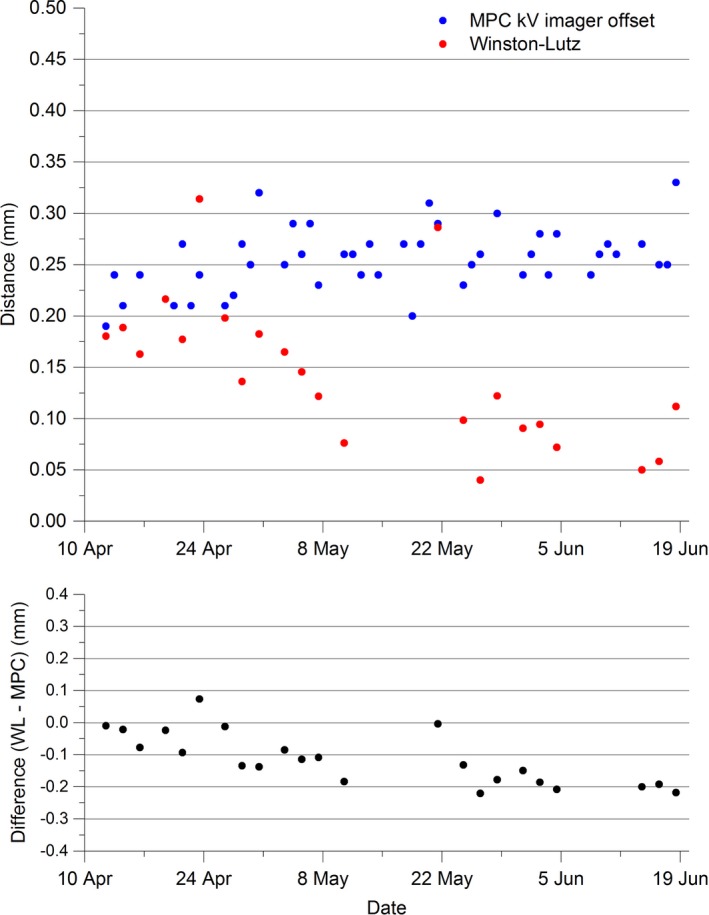
MPC kV imager offset and kV imager offset and in‐house Winston–Lutz distance between imaging and radiation isocenter.

The results of Fig. 2 demonstrate the agreement between the MPC kV imager offset and the in‐house Winston–Lutz‐measured coincidence of imaging and radiation isocenter. The Winston–Lutz result is generally smaller than MPC and appears to be trending lower while MPC appears stable. The maximum difference is 0.22 mm. The MPC mean is 0.26 ± 0.03 mm (1 SD) while the Winston–Lutz mean is 0.15 ± 0.10 mm (1 SD) confirming the Winston–Lutz results to be on average lower than MPC and less consistent. The data are not within statistical agreement based upon the t‐test [t(26) = −7.26, *P*<<0.001].

#### 3.B.3 Sensitivity to focal spot position change

**Figure 3:**
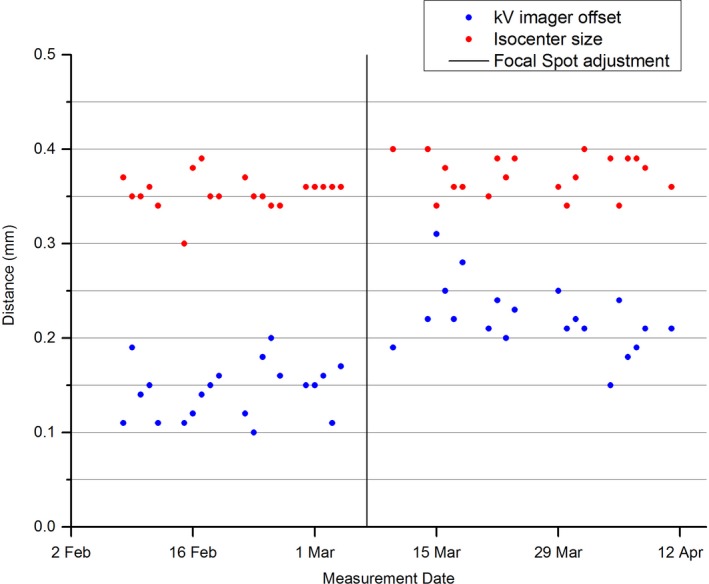
MPC isocenter size and kV imager offsets leading up to and following the focal spot adjustment

The results of Fig. 3 appear to show that the MPC isocenter size and kV imager offsets were relatively stable and constant before the focal spot adjustment. After the adjustment, the isocenter size appears unchanged, however, a systematic shift in the results is apparent for the kV imager offset results. The mean and standard deviation values for the isocenter size and kV imager offsets are presented both before and after the focal spot adjustment in Table 2 and the mean values were tested for statistical agreement using the t‐test. The t‐test shows that neither parameter was statistically equivalent before and after the focal spot position change [t(37) = 7.30, *P*<<0.0001 and t(38) = 2.99, *P* = <0.0049, respectively]. However, the t‐test results indicate a greater change in the kV offset parameter and hence greater sensitivity to the focal spot position change than the isocenter size parameter.

**Table 2:** MPC isocenter size and kV imager offset results before and after the focal spot position adjustment. [mean (mm) ± 1 Standard deviation]

MPC Parameter	Before adjustment (n = 20)	After adjustment (n = 20)
Isocenter size	0.35 ± 0.02	0.37 ± 0.02
kV Imager offsett	0.14 ± 0.03	0.22 ± 0.04

### 3.C Couch

The results of Table 3 show that the MPC couch lateral mean measurement agrees within three standard deviations of the mechanical QA measurements. MPC couch vertical and rotation agree within two standard deviations and couch longitudinal, pitch, and roll within one standard deviation.

**Table 3:** Comparison between MPC (n = 89) and mechanical QA (n = 7) couch tests. Difference from nominal. Mean ± 1 SD

Couch	MPC (mm/°)	Mechanical QA (mm/°)
Lateral	−0.16 ± 0.04	−0.04 ± 0.04
Longitudinal	0.02 ± 0.02	0.02 ± 0.04
Vertical	−0.02 ± 0.03	−0.08 ± 0.40
Rotation	−0.16 ± 0.01	−0.01 ± 0.07
Pitch	−0.01 ± 0.01	0.00 ± 0.09
Roll	−0.01 ± 0.01	−0.02 ± 0.10

#### 3.C.1 Sensitivity to miscalibration

For all couch axes a deliberate offset in the calibration was not detectable by MPC. An indirect exception to this was when the couch rotation calibration was offset by 0.5°. After this miscalibration the MPC rotation was unchanged within repeatability. However, the MPC couch lateral and longitudinal values changed by 0.49 mm and 0.44 mm, respectively.

The results of Table 4 show the MPC couch measurements after changes in the calibration span for the lateral, longitudinal, and vertical axes. The results show agreement between MPC and the expected value to within 0.04 mm.

**Table 4:** MPC measured changes in couch lateral, longitudinal, and vertical with deliberate miscalibration of the calibration span

	Expected change (mm)	MPC change (mm)	Difference (mm) (expected – MPC)
**Lateral**			
Larger span	−0.75	−0.76	0.01
Smaller span	0.75	0.71	0.04
**Longitudinal**			
Larger span	0.75	0.76	−0.01
Smaller span	−0.75	−0.79	0.04
**Vertical**			
Larger span	2.0	1.99	0.01
Smaller span	−2.0	−1.96	−0.04

### 3.D Sensitivity of MPC to Phantom tilt

Following the variation IN both phantom roll and phantom pitch none of the MPC parameters varied outside the measurement repeatability presented in Table 1.

## 4 Discussion

### 4.A Repeatability

The repeatability results of Table 1 are well inside the tolerances for all tests indicating that the tolerances are meaningful in that recorded fails are distinguishable from day to day variation.

### 4.B Isocenter

The statistical disagreement using the t‐test between MPC isocenter spheroid size and the in‐house Winston–Lutz does not suggest which method is more accurate. Excluding a single outlier in the Winston–Lutz data, the maximum disagreement between Winston–Lutz and MPC is at 0.11 mm, which is clinically insignificant.

The generally larger result of the kV imager offset compared to the Winston–Lutz method is not unexpected. The MPC kV imager offset provides the worse‐case difference in the projection of radiation isocenter spheroid to center of the kV imaging panel. This is a surrogate and hence not exactly the same as the distance between the radiation isocenter centroid and the kV imaging isocenter presented by the Winston–Lutz mean shift parameter. The IsoCal calibration shifts the kV imager panel with gantry angle so that the radiation and kV isocenters are coincident. Since the kV imager offset is measured after IsoCal corrections have been made then it provides a measure of the validity of the current IsoCal calibration. As such, the kV imager offset provides a surrogate for radiation and kV isocenter coincidence. If the user had a fail in an MPC kV imager offset measurement then this would be actioned in the first instance by performing IsoCal calibration.

The results of Table 2 and of the t‐test show that the kV imager offset parameter is sensitive to changes in beam focal spot position. This is expected as the effect of altering the beam focal spot is to shift the beam laterally and hence the projection of the radiation isocenter will shift relative to the imager center. Since the kV imager offset is primarily a measure of the correctness of the IsoCal calibration the results suggest that the IsoCal calibration should be performed after any focal spot position beam steering.

### 4.C Couch

The insensitivity of MPC to offsets introduced into the couch calibrations suggests that MPC is not suitable for testing couch absolute position. Such testing is not a recommendation of AAPM TG‐179. For IGRT purposes the accurate travel of the couch, which MPC tests, is more important than absolute position so that IGRT couch shifts can be performed accurately to place the patient in the correct treatment position.

The agreement between mechanical methods and MPC for the couch tests over the 4‐month period is heavily influenced by the accuracy of the mechanical methods. For the couch longitudinal, lateral, and vertical, the couch was shifted based upon the ruler/tape measure and the couch readout value was recorded. This allows results to be recorded to 0.01 mm resolution. However, the resolution limit from the ruler/tape measure was 0.5 mm. When calculated as a span scaled to the MPC measurement span this equates to a measurement resolution of 0.03 mm, 0.13 mm, and 0.75 mm for couch longitudinal, lateral, and vertical, respectively. The larger the measurement span the finer the measurement resolution is. This explains the relatively large standard deviation of Table 3 for the couch vertical mechanical results. The measurement resolutions suggest that for a more accurate evaluation of the MPC couch over the time period, a larger mechanical QA span should be used. However, the distances used in the departmental mechanical QA program were chosen to represent the standard clinical range and the aim of the study was to compare MPC to standard routine QA testing.

The results of Table 4 indicate that MPC is highly accurate to gross changes in couch calibration span. Such changes in couch calibration span simulate a mistake in user calibration or a fault in the encoder and the results indicate that MPC would detect such a problem accurately enough to alert the user to any significant fault. The analysis is limited to the lateral, longitudinal, and vertical axes by the fact that the couch pitch, roll, and rotation axes utilize single point calibrations and hence the calibration span could not be altered using the methods of this study. However, the measured change in couch longitudinal and lateral when there was an offset in the couch rotation calibration suggests that an observed change in these two values of similar magnitude might be able to be used to diagnose a couch rotation offset problem. However, the unexpected magnitude of the changes makes this process uncertain.

Even with the limitations to the couch comparison outlined, the greatest difference in mean values between MPC couch and mechanical QA over the 4‐month period measurements was 0.12 mm and 0.15°. These values are clinically insignificant. This result along with the highly accurate sensitivity of MPC to gross changes in span of MPC suggests that MPC is suitable for daily couch testing for IGRT considering the AAPM TG‐179 tolerance of ± 2 mm and the accuracy of the current alternatives available such as the Marker Match test.

### 4.D Sensitivity to phantom tilt

The lack of sensitivity of the MPC geometric tests to varying the phantom pitch and roll by one degree indicates that none of the tests are reliant on the accurate pitch and roll of the phantom. The MPC couch pitch and roll tests as well as the gantry‐relative tests are based upon the relative changes across multiple images of the phantom. As such, the pitch and roll of the phantom cancels out and does not affect the measurement.

## 5 Conclusion

For accurate IGRT the radiation isocenter size, coincidence of radiation isocenter with imaging isocenter and accuracy of couch shifts must all be accurately quantified. The MPC checks are adjudged to be accurate for radiation isocenter size and for couch shift accuracy. The kV imager offset parameter does not provide a direct measure of radiation to kV isocenter coincidence, but acts as a surrogate. However, if a fail in kV imager offset is recorded then redoing the IsoCal calibration is indicated. The IsoCal calibration should then improve alignment between the radiation and kV isocenter spheroids. For a daily test of isocenter alignment, the MPC kV imager offset should suffice and could be assured with a less frequent Winston–Lutz or Isocal verification measurement.

## Acknowledgment

The authors thank the Calvary Mater hospital Newcastle Radiation therapists for their MPC data collection. We would also particularly like to thank the head Biomedical engineer of the radiotherapy department Calvary Mater hospital Newcastle for his insights into linac operation and his assistance in safely miscalibrating the linac for the sensitivity tests.

## Conflict of interest

The authors declare no conflict of interest.
